# Navigating norms: a qualitative exploration of factors shaping contraceptive use in Senegal

**DOI:** 10.3389/fgwh.2025.1527733

**Published:** 2025-07-28

**Authors:** Manuela Reveiz, Rahmatoulah Gueye, Thaïs González Capella, Marieme Fall, Nour Horanieh, Elizabeth Larson, Beniamino Cislaghi

**Affiliations:** ^1^ Department of Global Health and Development, London School of Hygiene and Tropical Medicine, London, United Kingdom; ^2^ LARTES-IFAN, Research Laboratory on Economic and Social Transformation, Cheikh Anta Diop University, Dakar, Senegal; ^3^ Department of Family and Community Medicine, College of Medicine, King Saud University, Riyadh, Saudi Arabia; ^4^ Department of Population, Family and Reproductive Health, Johns Hopkins Bloomberg School of Public Health, Baltimore, MD, United States

**Keywords:** family planning, contraception, social norms, francophone West Africa, gender norms

## Abstract

**Introduction:**

Despite the increasing contraceptive, women in Senegal are facing both material and social obstacles to access family planning (FP) services. Decisions regarding contraceptive use involve an interplay of influencers, including the woman, her partner, family members, healthcare providers, and the social norms in place. This study employs social norms theory to explore how these dynamics shape women's contraceptive choices, examining the societal expectations, influential relationships, and strategies women use.

**Methods:**

This qualitative study involved 256 participants (130 men and 126 women) from four regions of Senegal (Dakar, Tambacounda, Ziguinchor, and Matam). We employed semi-structured interviews (116) and focus group discussions (16 FGDs with 8–10 participants each) to explore experiences related to family planning access and services. Participants were recruited through purposive sampling. Thematic analysis was performed using NVivo 12 Pro.

**Results:**

Participants highlighted how social norms on marriage, infertility, and childbearing heavily influence women's contraceptive use decisions. Participants also mentioned social norms that directly discouraged contraception use, labeling women who used it as disloyal or unloving. The failure to fulfill normative expectations resulted in various forms of sanctions. Given the normative system, numerous women opted to make decisions regarding childbearing in secrecy. Finally, a system of beliefs that participants held about religion and healthcare providers intersected with contraceptive utilization.

**Discussion:**

Our findings provide evidence of the importance of integrating social interventions into FP interventions to help reframe social relations. Three policy implications arise: (1) Addressing broader social needs and support mechanisms. (2) Integrating insights from violence against women research and theories on the dynamics of abuse into FP programs. (3) Integrating men further into FP programming to address misconceptions.

**Conclusion:**

Drawing on data from 256 young people, men, and women of reproductive age, we uncovered how women navigate the terrain of social norms within their networks, shaping their choices concerning contraceptive utilization.

## Introduction

When a woman chooses to use contraception, the consequences she faces can be influenced by a complex interplay of factors. Senegalese women are increasingly using more contraceptives. Between 2013 and 2023, the contraceptive prevalence rate increased from 16.6% to 29.4% among women aged 15–49 in formal or informal marital unions (1); understandably, in the same period, the number of 15–49 married women with unmet family planning needs declined (from 30.2 to 22.4%) (1).

Despite these recent increases in their uptake of modern contraceptives, women in Senegal are facing both material and social barriers to accessing family planning services. Material inequities related to geographical, economic, and education-related factors in accessing services and methods exist. Greater access to contraceptives is observed among married women in urban areas, with 33% using contraceptives compared to 17% in rural areas (2); among women in the highest economic quantile (36%) than those in the lowest quantile (16%); and among women secondary or higher education (33%) than those with no education (16%) (2). Other discriminatory variables for contraceptive use include mean age at marriage, access to qualified Health Care Providers (HCP), and discussing use with a partner (3–9).

Senegalese women also face social obstacles when accessing contraceptives. In Senegal, contraception is sometimes accepted for married women but still heavily critiqued for unmarried women (9, 10). Decisions about who can access it and who has the final decision on utilization are influenced by a combination of factors, including the woman, her husband, her family, healthcare providers, and prevailing social norms (6, 9, 11, 12). According to social norms theory, social norms are unwritten rules dictating what actions are socially appropriate (or not) for people within a given group (13–15). Contravening social norms can lead to negative sanctions (16). These sanctions can include gossiping, isolation, or even violence.

Across Sub-Saharan Africa, a growing body of literature explores how social norms shape contraceptive behaviors and decision-making. In Niger, Mayaki et al. examined the role of traditional values and norms in shaping family planning practices and found that social norms negatively affected contraceptive use among urban women without schooling, while women with schooling were less influenced by those norms (17). Findings from Kenya highlight gendered differences in how social norms and networks can shape contraceptive use: Lahiri et al. found that pro-modern contraceptive norms use among peers was positively associated with modern contraceptive use among women, while no significant association was found among men (18). Among this group, engagement tends to remain limited due to factors such as misinformation, disapproval of unilateral decisions by women to use family planning, lack of male-targeted services, and gaps between perceived community norms and actual communication and involvement (19, 20).

Despite a growing evidence base, few recent qualitative studies in Francophone West Africa have explored how social norms and networks influence contraceptive decision-making, particularly those that include diverse male and female perspectives across age groups (19, 20). This study will draw on social norms theory to explain how social norms impact women's access to contraception by focusing on four key areas: the societal beliefs and expectations that shape women's choices, the significant influencers who weigh into their decision-making process, the strategies women use to navigate challenges when seeking contraception, and other factual beliefs affecting women's use of contraceptives, influencing whether they pursue or forgo contraceptive use.

## Methods

### Participants

The study involved 130 men and 126 women and was conducted across four regions of Senegal: Dakar, Tambacounda, Ziguinchor, and Matam, representing urban, peri-urban, suburban, and rural areas, respectively.

### Study design

Qualitative methods were used to examine the experiences of women and men with family planning, in relation to access, use, experiences and feelings towards family planning services in Senegal. We used two qualitative research methods: semi-structured interviews and focus group discussions We conducted a qualitative study with a total of 256 men and women of reproductive age (16–49). We conducted 116 semi-structured interviews, and 16 focus group discussions (FGDs) with 8–10 participants each, totaling 140 participants in FGDs. Focus group discussions were divided by gender and age, with participants categorized as either below 25 or above 25 years old. All interviews were recorded after obtaining written and verbal consent. An initial interview guide was developed based on a previous literature review, and workshops conducted with FP professionals working in program implementation, including healthcare providers, government officials and NGO workers in Francophone West Africa (FWA).

### Sampling

We adopted a purposive sampling strategy to recruit participants according to the following inclusion criteria: female and male individuals who were current users of family planning services or had previously been exposed to family planning services in Senegal. We identified key community members, including community health workers and local leaders, to facilitate the recruitment process. Additionally, we used social media to identify further participants. Refreshments were offered to participants during the focus groups.

### Data collection

Data were collected either in Wolof, Pulaar (Fula), or Jola by local researchers, and later transcribed and translated into French by local transcribers. To prepare for data collection for the qualitative study, our team recruited 16 Senegalese local researchers with a background in qualitative research methodologies to conduct the data collection. We conducted a five-day workshop with the Senegalese researchers to adapt our qualitative data collection methodology and tools. During the workshop, the researchers shared their views of the preliminary topic guide questions and methods. Several participatory activities were conducted with the researchers including, role-playing, practice interviews, and focus groups. Training on acquiring consent was also provided to researchers. Data collection was conducted immediately after the workshop and both local and London School of Hygiene and Tropical Medicine's ethics approvals were obtained. Each interview lasted approximately 1 h. Focus groups lasted approximately 3 h, including a break.

### Analysis

Thematic analysis was conducted with the support of NVivo 12 Pro Software (QSR International Pty Ltd). Data analysis was iterative: initially, one researcher (MR) open-coded a subset of the data and developed an initial set of hierarchical labels using grounded theory (21). Themes and subthemes were established according to the most prevalent concepts that emerged during the inductive process and the theoretical input of a fourth researcher. Three researchers coded a set of interviews and cross-verified all codes to guarantee consistency of the coding framework then applied thematic analysis with support of the selected software. This process was repeated until the meaning of all labels which were agreed upon by all researchers. All researchers coded data from all regions. The coding framework was revised and expanded as new themes and subthemes appeared until exhaustiveness was reached. Axial coding was conducted at a participatory workshop in Dakar in collaboration with a subset of the same researchers who had previously conducted the interviews and focus groups across regions.

## Results

Our research focuses on four key areas: the societal beliefs and expectations that shape women's choices, the significant influencers who weigh into their decision-making process, the strategies women adopt to navigate challenges when seeking contraception, and other factual beliefs affecting women's use of contraceptives.

### Prevailing social norms affecting family planning practices

Within the system of social norms, the first theme explores expectations surrounding marriage and childbearing, shaping when and how women should marry and have children. The second theme addresses norms that influence family planning, guiding women's decisions about contraception.

### Social norms of marriage and childbearing

Findings from our study reveal that social norms on marriage and childbearing profoundly influence women's decisions regarding family planning and contraception use. Women often feared being blamed or judged by their partners if they did not fulfill expectations to bear children. This pressure, driven by the desire for children from both partners and extended family, can strongly influence women's decisions regarding contraception use. Unions without offspring were regarded as lacking purpose by most of our participants. A man in Tambacounda shared that “*you always expect a child when someone gets married. If you go for a year without having a child, you start making judgments about the couple*” (Focus Group, Adult Man, Tambacounda). Although it was more often mentioned among female participants, some male participants also experienced pressure from social networks. In Matam, male participants explained that men try to have children right after marriage “*because this is what shows whether the man is sexually powerful*” (Focus Group, Young Man, Matam). There are social repercussions because “*Married men who do not have children are frowned upon*”. Male participants reported incidents of verbal harassment due to childlessness when married and without children. While some participants framed it as a consequence of the wife's infertility “*The husband may be the victim of insults from his parents who put pressure on him as to why your wife is not able to procreate*” (Focus Group, Young Man, Matam), an overwhelming majority referenced their manhood being contested if a man was unable to conceive. A man explained that “*When you get married and you go for 4 or 5 years without having a child, people will wonder. They think, is this one really a man?*” (Interview, Adult Man, Ziguinchor). Male participants from all regions employed language that perpetuated masculine gender stereotypes, with phrases such as “*real man*” being commonly used in all regions. These comments were identified as hurtful:

“The fact that they tell you that you are not a man because you don’t have children can hurt you to such an extent that if your wife told you that she does not want to have children yet, you will put the pressure of wanting to have a child because being treated like a subhuman doesn't suit you.” (Focus Group, Young Man, Matam).

In discussions about childbearing, women recounted instances where women in their community faced unjust blame for infertility. In focus groups in Zinginchor, Matam Tambacounda, female participants agreed that “*Having a child is God's responsibility and most of the time women are blamed and thought to be the ones who can't have children. But sometimes it might be the son's fault*” (Focus Group, Young Woman, Zinginchor). A lack of offspring was often blamed on women “*without proof*” because “*There are realities that do not depend directly on the wife. They go so far as to call the wife a man in order to hurt her*” (Focus Group, Adult Woman, Matam). In contrast, only a few participants from Dakar had different perspectives. A woman from Dakar shared that “*sisters-in-law think that you must have a child when they should know that the purpose of marriage is not to have children*” (Interview, Adult Woman, Dakar), and a man from Dakar argued that “*the child is not part of a marriage contract, it is not an obligation*” (Interview, Adult Man, Dakar).

In some rare instances, participants described situations in which verbal harassment took place when couples had “*too many children*”. Some of our participants also shared that if the woman has too many children, she can be frowned upon by those around her. This is confirmed by this young woman who explained that “*if the person frequently has children, people will start saying ‘this person is just having children, she looks like a goat*’”(Interview, Young Woman, Matam). A man illustrated this situation as following (Interview, Adult Man, Ziguinchor):

“The way she has close births, her friends who are outside can say that: she has her babies all the time, like a “goat” or a “chicken”. Now, it hurts her when she hears that, but she won't be able to react because she will say to herself that she can't do anything about it because every time my husband needs me (sexually), I can't refuse him because that’s why I'm here today. So I can't refuse because if I refuse, he can create problems for me and it’s not worth it.”

### Social norms influencing family planning use

Across interviews and focus groups, we found evidence that a network of normative beliefs strongly impacted women's decisions. Beyond the expectation to have children within a family unit, participants mentioned social norms that directly discouraged contraception use, labeling women who use it as disloyal. Married women who utilized contraception were often viewed as being unfaithful to their husbands or lacking affection towards them. Many male participants believed its use may signify a “lack of love” within a marriage. When asked what he would do if his wife used contraception in secret, a man from Tambacounda expressed that he would “*divorce her in front of her parents because she doesn't love me. If she loved me she wouldn't hide it from me. A man must not be weak*” (Interview, Adult Man, Tambacounda) and a man from Matam expressed that “*Most married women who do it, do it either because they don*’*t love their husbands enough or because it's a forced marriage or a marriage of convenience*”. To mitigate challenging circumstances, some women opt to secretly use these methods despite the prohibition set by their husbands. A man from Dakar explained how women can use contraception as a tool to defend themselves in case a marriage goes awry:

“If we see a woman doing planning it is because she does not love her husband, she will look if the couple is suitable for her or not, if it is suitable for her she will remove the planning if it is the opposite she will get rid of the marriage without having children” (Interview, Adult Man, Dakar).

Many male participants, from all age groups and in all regions stigmatized and labeled unmarried women that use contraception as prostitutes. Indeed, the use of contraception was commonly perceived as synonymous with promiscuous and immoral behavior. A woman from Dakar explained that “*prostitutes and others who can be categorized as prostitutes do Family Planning*.” (Interview, Adult Woman 8, Dakar). A young woman from Matam explained that prostitutes are viewed as “*someone who is used to doing these kinds of acts*”, independent of whether such acts were monetized or not (Interview, Young Woman 4, Matam). Finally, we found in our data evidence of exceptional cases in which deviance from the norm was possible. Participants believed that imposing contraception was a way “*to protect people with mental illnesses*.” Indeed, a young man from Dakar suggested that “*we do family planning for people who are mentally ill and who can sometimes be raped*”. Parents were the primary source of family planning decision-making, as highlighted by a young man from Matam who explained that “*Parents are obliged to do family planning because these women are not mentally stable and you can never trust men as they are sexual predators*.” (Interview, Young Man 1, Matam). When questioned about the presence of unrequested family planning advice at healthcare facilities, a young woman from Dakar shared:

“Yes, it happened once with a girl who had psychological problems. She is an adult whose family cannot take care of her. That’s why they put her on contraception to prevent her from getting pregnant” (Interview, Young Woman, Dakar).

### Key influencers and potential sanctions in women's family planning decisions

Key influencers in women's family planning decisions include family members, partners, and the broader community. These groups can shape women's contraceptive choices through potential social sanctions, either offering support or imposing restrictions based on prevailing norms.

### Family

Participants identified various circles within women's social networks that played a crucial role in upholding and enforcing normative beliefs. Reactions from these different reference groups significantly impacted women's family planning decisions. The failure to fulfill normative expectations resulted in various forms of sanctions perpetrated by the couple's social network. One of the reference groups frequently mentioned as contributing to upholding a system of norms was family members, particularly mothers-in-law and sisters-in-law. One young woman from Dakar specified that “*This is why when you are in a household and you go a long time without having children, your mother-in-law tends to make life hard for you*” (Focus Group, Young Woman, Dakar), and another participant shared that “*The mothers-in-law go so far as to make slanderous remarks. Women in this situation are victims of suffering*” (Focus Group, Adult Woman, Ziguinchor). Female participants also identified sisters-in-law as an important source of psychological distress. A participant shared that sisters-in-law “*are the first to denigrate you in the eyes of people, especially when you cannot have children*” (Interview, Adult Woman, Dakar). A woman from Dakar recounted how wives can be subjected to unequal treatment and comparison based on the number of children they have:

“This happened to me once when I was with my co-wife. We came to the house at the same time and when I had a child and she didn't, my mother-in-law called her names. She also told her that she could go back if she can't have children” (Focus Group, Adult Woman, Dakar).

Another young female participant from Tambacounda described how mothers-in-law sometimes had a stronger influence on a woman's family planning decision than the husband, especially if the husband “*is a weak person*” (Interview, Young Woman, Tambacounda). At times, these sanctions in the form of psychological or verbal aggressions escalated to include excessive housework demands from in-laws, placing added physical pressure on the wife until she became a mother. Given this situation, several women reported covert use of contraception. For instance, an adult woman from Dakar explained “*mother-in-law often [..] urges me not to do family planning. If I told her the truth, she would get mad at me and tell me to take it off*” (Interview, Adult Woman, Dakar).

### Partner

A second category of people frequently mentioned by participants were partners. Partners also played a key role in upholding norms and at times-imposed sanctions if women did not uphold normative expectations.

Participants provided evidence of the potential harm that might come from discussing reproductive desires with their spouses. One, for instance, said that discussing those desires might “*bring many problems*” (Interview, Young Man, Dakar). The main reason for these misunderstandings, participants suggested, is that a man and woman “*will never find a consensus*” (Focus Group, Young Man, Matam), and the disagreement might be as deep as to lead to divorce or physical violence. Male participants were uncomfortable if their wives used contraception while they were traveling out of the city, fearing that their wives would commit adultery. A male participant from Dakar shared that after he came back home from traveling, he asked his wife if she had removed her family planning method of choice: “*She replied that she had taken it off, but she lied. I shook her and got angry. She confessed no, she hadn't taken it off. I told her: if I come home next month, and you haven't taken it off yet, it'll be you and me in this house*” (Interview, Adult Man 1, Dakar). Some participants explained how contraception can be used to protect oneself in case of abandonment within a marriage “*Some women do* [covert contraception use] *because they are afraid of having many children and that one day the man will repudiate them and leave them alone with the children and problems*” (Focus Group, Young Man, Matam).

### Community

Furthermore, our participants provided evidence that the community in which the couple is situated also imposes normative expectations regarding childbearing within a marriage. Women who had publicly utilized contraception were frequently subjected to blame and accusations of infertility if they were unable to conceive soon after discontinuing its use. In a focus group with adult women, a mother from Matam shared that “*It can also happen that when you are ready to take* [the implant] *away you are told that you cannot have any more children. So family planning can make you lose any respect from your family*” (Interview, Adult Woman, Matam). Another participant shared “*Planning is breaking up a lot of marriages because [..] once you get planning out, you won't have children*” (Interview, Adult Woman, Ziguinchor). Many participants reported believing that family planning led to infertility. Women who chose to secretly utilize modern contraception methods were often seen as unable to successfully carry a pregnancy to term by their community, resulting in sanctions such as exclusion. Despite these norms and reactions against contraceptives, our findings reveal that many women still utilize family planning methods. A woman from Matam shared that the reason why she hides contraceptive utilization from her social network is because “*I don*’*t want to suffer through pregnancies. They are not at home and are unaware of my suffering*.” (Interview, Woman, Matam).

### Women strategies for concealed use of contraceptives

Given the prevailing normative system and the accompanying social repercussions, numerous women opted to make decisions regarding childbearing in secrecy from their spouses. One young man said that a woman might be “*afraid that the subject will create problems for her in his relationship*” (Focus Group, Young Man, Matam). Several participants mentioned women might also fear their husbands. One, for instance, mentioned that a woman might “*be afraid to tell her husband* or *lack trust in her because he is impulsive*” (Interview, Young Woman, Tambacounda). In these cases, women might prefer to hide contraception utilization from their husbands.

Woman from Matam: “I didn't tell him how many children I want, but he, on the other hand, told me he wants to have up to ten children.”

Interviewer: “Why didn't you tell him what you want?”

Woman from Matam: “Because he wants a number that doesn't suit me, and he is against family planning. I don't want him to realize that I am practicing family planning. If I tell him that I want to stop at six children, he will know it's because of family planning. But if I don't tell him, he might think it's just divine will.”

Some women opted to initiate a discussion with their partners but, unable to secure approval from their husbands, resorted to using contraceptive methods without their knowledge, arguing this was the only way to safeguard their health.

“Yes, because it’s the woman who knows what is good or not for her. Men only think about having children; it’s the women who know the pain of pregnancy and childbirth. That’s why I hid my decision from him to avoid any problems between us.” (Focus group, Woman, Matam)

“you know also it is necessary that women do it in secret because some men do not know how to do anything in their life but to have sex and have children, so if the woman knows that she cannot continue at this rate she can hide [family planning] to just rest” (Focus Group, Young Woman, Tambacounda).

Women's concealment of contraceptive use often led to anxiety, with fears of consequences in case the husband found out about its use “*Sometimes I would get goosebumps because I didn't tell him.” “Family planning can create misunderstandings within the couple. The worst can happen when the husband is not informed. This can create problems in the relationship, potentially leading to divorce*” (Focus Group, Women, Ziguinchor). As a result, some women followed their husband's authority by not using any modern method. This is the case for a woman from Dakar who feels the need to rest her body after giving birth and wants to use contraceptive methods but decides to follow her husband's desires over her own to avoid problems in her household. She affirms: “*If there is a conflict about how many children you want to have in a couple, you always have to comply with your husband's decision and do what he wants.*” (Interview, Adult Woman, Dakar).

Finally, participants revealed that some couples collaborated to undertake birth spacing and preferred to conceal their contraceptive utilization from their family to avoid confrontation. One woman shared that only she and her husband knew about her contraceptive use because her husband had asked “*to keep it between the two of us and not let the rest of the family know*” (Interview, Adult Woman, Dakar). In another interview in Dakar, a woman shared that:

“If the woman wants to work. Her husband can support her without the rest of the family knowing what is really happening behind the scenes. However, the family might cause her problems without even knowing that she has her husband’s approval and support.” (Interview, Adult Woman, Dakar).

### Other factual beliefs affecting women's use of contraceptives

A system of beliefs that participants held about contraception, religion, and healthcare providers intersected with their decision-making, influencing their choices on contraceptive utilization. Our findings indicate that men often play a determining role in the women's continuation and discontinuation of contraception, which might lead to covert contraception use by women. An overwhelming majority of our participants believed that women should not use contraception without their husband's consent. The most common reason to prohibit its use included differences in opinion regarding the desired number of children between couples and negative beliefs about the consequences of contraceptive methods. Female participants were more familiar with reproductive health services and exhibited less skepticism towards doctors and midwives compared to their spouses. Despite the availability of services, male individuals exhibited skepticism towards healthcare professionals, leading to a limited utilization of family planning services: A female participant from Dakar explained:

“I have talked to him [husband] about family planning but he often retorts by telling me that family planning is just a scam by health care providers and that it has consequences for women’s health. I let him know that [..] a woman who gives birth often is vulnerable and can catch diseases” (Interview, Young Woman, Dakar).

Many participants were adamant that they do not want to use contraception because of their religion, as is the case of a man from Matam who explains that women do it discreetly because: “*religion does not allow family planning and as a Muslim, a man will never allow his wife to do family planning*.” (Interview, Adult Man 6, Matam). Male participants explained that, in the context of Islam, the responsibility of men toward their wives is of paramount significance. This is intricately linked to their stance on contraception use. Many male participants from all regions expressed concerns about its adverse effects on their wives' health. A man from Matam explained that “*For me, I will refuse because she may get sick from family planning and it will be my responsibility.” (Interview, Adult Man 3 Matam).* However, a few male interviewees mentioned that contraception is essential to preserve women's health and therefore it does not contradict Islam. A man from Tambacounda explained that “*planning is good for me because I have had four children, but my wife often has complications during pregnancy and has even had surgery. I have discussed this with my in-laws and my family so that my wife can access family planning services*.” (Interview, Adult Man 1, Tambacounda). Another man from Dakar explained that *“Islam said that if you have three children and you risk your life if you have a fourth child, Islam allows you to do family planning” (Focus Group, Adult Man 6, Dakar).*

## Discussion

Our findings extend the scope of research on the dynamics between social norms and contraceptive use. We unraveled a delicate balancing act undertaken by women as they navigate the complex terrain of family planning decision-making. While some women covertly employed contraceptive methods to assert their agency, societal norms often restricted their agency as a consequence of this covert use, labeling these women as infertile or immoral. The couple's agency over their reproductive choices was frequently restricted by societal expectations, perpetuating cycles of secrecy. Decisions related to family planning often favored the perspectives of men over women. Our findings also highlight the importance of acknowledging the intersectionality of factors, such as mental illness, when it comes to informed choice of contraceptives (22, 23).

Our findings have three major implications for policy and practice: (1) Addressing broader social needs and support mechanisms. (2) Integrating insights from violence against women research and theories on the dynamics of abuse into family planning programs. (3) Integrating men further into family planning programming to address misconceptions.

Our findings provide evidence of the importance of integrating social interventions into family planning interventions to help reframe social relations. Our findings indicate that while contraceptive methods were widely available at healthcare facilities and healthcare providers were generally trusted by women, men remained hesitant. This hesitancy, coupled with prevailing social norms around decision-making and some women's concerns about infertility, illustrates that merely ensuring physical access was insufficient to guarantee access among women with unmet need. Medicalization has been used to describe the process of defining and treating certain conditions, behaviors, and medical issues. The medicalization of family planning in Francophone West Africa has contributed to the improvement of reproductive healthcare, averting maternal and neonatal mortality and morbidity and offering women greater control over their reproductive health despite persistent disparities between urban and rural regions in Senegal (24–26). The medicalization of family planning has been criticized as well in the literature as a source of detachment between the patient's normative context and the doctor (27, 28). Our findings provide evidence that the medicalized nature of family planning services, while empowering women, may inadvertently pose a barrier to open communication regarding social norms (29–31). Our research indicates that effective interventions must not only improve contraceptive access in health facilities but also address women's social and contextual needs.

Violence against women often stems from a process where the decisions of a woman's partner are imposed on her, highlighting how social norms that center control and dominance in decision-making play a critical role in perpetuating violence. Our findings indicate that instances of physical abuse directly linked to family planning practices were rare. However, the data revealed strong connections between social norms, sanctions, and family planning use. These sanctions often manifested as verbal abuse, social exclusion, and threats. Violence against women remains a persistent challenge in Senegal. About 27% of women in Senegal aged 15–49 have suffered physical violence since the age of 15, and 24% have experienced IPV (32, 33). Understanding the complex decision-making process of contraceptive use is crucial, as it can both influence and be influenced by violence against women. Violence can lead directly to unwanted pregnancy through sexual assault or reproductive coercion (34–36). Violence can be perpetrated regardless of the relationship between the victim and the perpetrator, and it can manifest itself in several forms, including coercion, physical, sexual, verbal, psychological, or economic harm, and it includes threats of violent acts as well (37). Participants reported experiencing some of the aforementioned forms of violence, with psychological and verbal abuse being largely the most common in connection with family planning decisions. We observed a connection between women's family planning decisions and the reactions of female figures within the community, notably mothers-in-law and sisters-in-law. These women held considerable influence in shaping women's choices and often endorsed normative expectations regarding childbearing. Our findings resonate with existing literature on violence against women in West Africa that studied the role of female social networks in shaping women's experiences of violence (38–40). J. Gupta and colleagues found that over 27% of women in Côte d'Ivoire reported in-law abuse, which was significantly linked to reproductive control by in-laws. C.A. Akurugu observed that in rural northern Ghana, woman-to-woman violence was common and often discussed and joked about, though it primarily took psychological rather than physical forms. Consistent with C.A. Akurugu's research, we found that violence against women manifested through sanctions in the form of psychological violence related to normative expectations about childbearing and marriage (38, 39).

Among our interviewees, one key obstacle raised was men's physical absence from healthcare visits and consultations, along with their social detachment from family planning matters. In Senegal, men are generally considered to be the head of the household and main protectors of the family, including of their wives (41). Our male participants shared a strong sense of responsibility toward their wives and the importance of caring for their partner's health within the context of following the teachings of the Islamic faith. Islam encompasses a holistic view of health, seeing it as a balance between physical and mental well-being (42). An often-overlooked reason in the literature for men's aversion to family planning is their perception that it harms women's health thereby conflicting with their duty to protect their wives. As portrayed in our research and reflected in the literature, men's intentions are frequently informed by incomplete medical knowledge (43, 44). While the desire to increase the number of children within a couple is a known and significant factor in opposing contraceptive use (7, 45) and was also the most prevalent factor in our study, another underexplored reason is the intention to shield their wives from the perceived harmful effects of contraception (e.g., side effects, heavy bleeding, perceived infertility, and sickness). Our qualitative findings suggest that husbands' actions can stem from a genuine desire to help their wives, but are also influenced by a lack of willingness to seek information at the health facility and accept alternative views on contraceptive methods.

These beliefs are also kept in place by a system of norms and social networks that connect family planning to harmful consequences threatening masculinity and power (46, 47). Our findings show that men see themselves as the head of the household with final decision-making authority and are strongly attached to this belief. Some men were also profoundly attached to their relationships and saw the possibility of being in a loving relationship threatened by the belief that their wife was going to engage in adultery by using family planning methods. In their study, Speizer and colleagues found that men's exposure to family planning programming—such as favorable messages from religious leaders, family planning content on television and radio, and community outreach activities—was associated with increased modern family planning use and discussions about family planning with their partners in four urban sites in Senegal (11). Our participants frequently cited the Bajenu Gox as a valuable resource for facilitating such conversations between couples and as a source of knowledge. The introduction of couple counseling within healthcare institutions was a solution recommended by our participants. Other similar interventions have employed formal written invitations to encourage male participation in women's reproductive care at healthcare facilities (48–51). The success of these approaches varies in the literature, ranging from not statistically significant, as observed by Stefanie Theuring and colleagues in a controlled intervention trial in Mbeya, Tanzania, to highly effective and significant, as reported by Theresa M. Exner and colleagues in a controlled trial in Nigeria, as well as by Bright Chigbu and colleagues's work in Nigeria as well (52–55). This approach recognizes the influential role of healthcare providers in shaping decision-making dynamics within couples to understand their social needs in the context of family planning (56–62).

## Limitations

The study ensures a diverse representation by including various age groups, genders, and regions, though bias might be introduced as the sample selected is based on individuals who are current family planning users or have been exposed to family planning programs. The study was conducted in four regions of Senegal covering a range of urban, suburban, rural, and peri-urban areas. Discussions were conducted in multiple languages to obtain a diverse sample, which could introduce translation biases. Additionally, the potential for social desirability bias in participants' responses, particularly in focus group discussions, should be recognized. Despite extensive training workshops, local researchers might introduce bias during the data collection phase, and researchers might introduce bias during the data analysis phase. The research team adopted a collaborative approach to data analysis, involving multiple researchers and conducting blind coding and cross-verification to reduce individual biases. Although our findings indicate that instances of physical abuse directly linked to family planning practices were rare, this could be due to an unwillingness among participants to discuss such sensitive issues.

## Conclusion

Drawing on data from 256 young people, men, and women of reproductive age, we uncovered how women navigate the terrain of social norms within their networks, shaping their choices concerning contraceptive utilization. This study shows women covertly employ contraception to maintain their health and well-being. In this context, female figures in the household emerge as potent agents, exerting societal pressure on men and women to conform to normative ideals of family size. Male participants exhibited a lack of willingness to seek information at the health facility and accept alternative views on contraceptive methods. Our findings expand the understanding of how social norms influence contraceptive use. While some women covertly assert their agency through contraception, societal norms often stigmatize them as infertile or immoral, restricting individual and couple autonomy. We found that while access to contraception has improved in healthcare settings, broader social needs remain unmet, particularly concerning violence against women, men's involvement at the facility level, and the influence of female family figures. Policy implications include addressing social norms, integrating violence prevention into family planning programs, and encouraging male involvement.

## Data Availability

The raw data supporting the conclusions of this article will be made available by the authors, without undue reservation.
